# Molecular markers associated with outcome and metastasis in human pancreatic cancer

**DOI:** 10.1186/1756-9966-31-68

**Published:** 2012-08-27

**Authors:** Anke Van den Broeck, Hugo Vankelecom, Rudy Van Eijsden, Olivier Govaere, Baki Topal

**Affiliations:** 1 Department of Abdominal Surgery, University Hospitals Leuven, Leuven, Belgium; 2 Laboratory of Tissue Plasticity, Research Unit of Embryo and Stem Cells, Department of Development & Regeneration, University of Leuven (KU Leuven), Leuven, Belgium; 3 VIB Genomics Core, University of Leuven (KU Leuven), Leuven, Belgium; 4 Department of Pathology, University Hospitals Leuven, Leuven, Belgium

**Keywords:** Pancreatic cancer, Surgery, Biomarker

## Abstract

**Background:**

Pancreatic ductal adenocarcinoma (PDAC) is a heterogeneous cancer in which differences in survival rates might be related to a variety in gene expression profiles. Although the molecular biology of PDAC begins to be revealed, genes or pathways that specifically drive tumour progression or metastasis are not well understood.

**Methods:**

We performed microarray analyses on whole-tumour samples of 2 human PDAC subpopulations with similar clinicopathological features, but extremely distinct survival rates after potentially curative surgery, i.e. good outcome (OS and DFS > 50 months, n = 7) *versus* bad outcome (OS < 19 months and DFS < 7 months, n = 10). Additionally, liver- and peritoneal metastases were analysed and compared to primary cancer tissue (n = 11).

**Results:**

The integrin and ephrin receptor families were upregulated in all PDAC samples, irrespective of outcome, supporting an important role of the interaction between pancreatic cancer cells and the surrounding desmoplastic reaction in tumorigenesis and cancer progression. Moreover, some components such as *ITGB1* and *EPHA2* were upregulated in PDAC samples with a poor outcome, Additionally, overexpression of the non-canonical Wnt/β-catenin pathway and EMT genes in PDAC samples with bad *versus* good outcome suggests their contribution to the invasiveness of pancreatic cancer, with *β-catenin* being also highly upregulated in metastatic tissue.

**Conclusions:**

Components of the integrin and ephrin pathways and EMT related genes, might serve as molecular markers in pancreatic cancer as their expression seems to be related with prognosis.

## Background

Pancreatic ductal adenocarcinoma (PDAC) remains a major cause of cancer related death, despite advances in surgical and medical care [1]. The majority of patients present with locally advanced or metastatic disease and die within 6–12 months. Even in the selected group of prognostic favourable localized and resectable PDAC, the 5-year overall survival (OS) is only 10-25% as the majority of patients develop disease relapse within two years after potentially curative treatment [2]. Additionally, the effect of systemic chemotherapy, either in adjuvant or in palliative setting, is low [3].

Although some parameters are described to be prognostic factors after curative surgery, such as lymph node and resection margin status, none has been consistently related to overall survival [4,5]. Moreover, even in patients with similar clinicopathological parameters, a wide range of survival rates is observed postoperatively [2]. This heterogeneous biology of pancreatic cancer and possibly related diverse response to treatment might be explained by differences in gene expression profiles. At present, molecular characteristics of PDAC carcinogenesis become gradually unravelled, but genes or pathways that specifically drive tumour progression or metastasis are not well understood [6,7]. Some studies have already linked gene expression profiles with lymph node status or advanced PDAC stage, but results are inconsistent [8-10]. Recently, a gene signature that subdivides PDAC in 3 subtypes was developed based on gene expression from microdissected PDAC material and cell lines. This signature would have a prognostic value and would be predictive for drug responses [11]. Microdissected material and cell lines however do not comprise the complexity of pancreatic cancer. PDAC is characterized by an abundant desmoplastic reaction that has long been ignored, but is now known to play an important role in PDAC tumorigenesis and progression [12,13].

Therefore, the aim of the present study was to define molecular characteristics related to pancreatic cancer progression, based on whole genome expression profiling of 2 human PDAC subgroups with similar clinicopathological features, but with extremely distinct survival rates after curative surgery. Additionally, we tried to gain more insight in the metastatic process of PDAC by comparing gene expression profiles of liver- and peritoneal metastases with that of primary tumour samples.

## Methods

### Primary PDAC and metastatic samples

Patients who underwent surgical treatment for PDAC between 1998 and 2008 were studied. Immediately after surgical removal of the resection specimen, a small part of the tissue was snap-frozen in liquid nitrogen and stored at −80°C; the other part was fixed in 6% formol and embedded in paraffin for histological examination. From patients with metastatic disease undergoing palliative surgery, core biopsies of the primary tumour and of liver (LM)/peritoneal (PM) metastases were taken and processed in a similar way. Haematoxylin-Eosin (H&E) staining was performed on each sample for histopathological confirmation according to the World Health Organization criteria. The study was approved by the KU Leuven ethical committee prior to patient recruitment, and received the study number ML3452.

Clinical and histopathological data from all patients were registered in a prospective database. Disease recurrence was defined as local or distant recurrence, diagnosed on follow-up imaging, performed routinely or because of elevated serum tumour markers.

### Classification of PDAC with good or bad outcome

**Figure 1:**
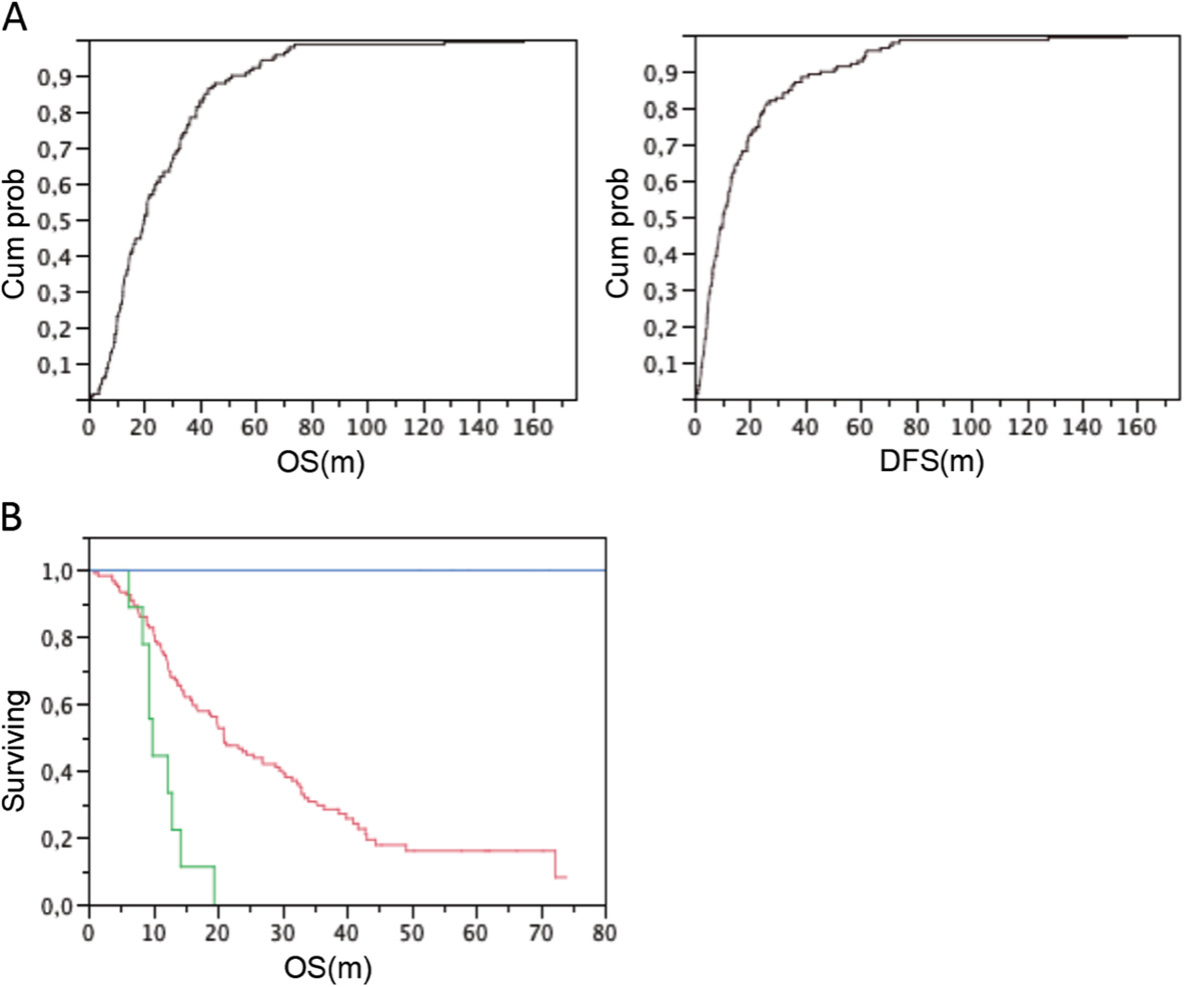
**Classification of PDAC patients based on outcome data.** (**A**) Cumulative curve for overall survival (OS, left) and disease-free survival (DFS, right), based on survival data of all PDAC patients with representative snap-frozen material. (**B**) Kaplan-Meier overall survival curve of patients respectively from the ‘Good’ (blue) and ‘Bad’ (green) outcome group, in comparison with the non-classified patients (red).

One hundred fifty-five patients suffering from PDAC were operated with curative intent. Postoperative follow-up was complete and closed in December 2011. Survival curves were determined using the Kaplan-Meier life-table technique. The median overall (OS) and disease-free survival (DFS) was respectively 22.3 months (95% confidence interval (CI) 18.7-29.0 m) and 12.0 months (CI: 9.0-13.3 m). None of these patients received pre-operative or neo-adjuvant treatment. Postoperative chemotherapy (n = 69) or chemoradiation (n = 29) did not influence OS or DFS in this patient group. Based on cumulative OS and DFS probability plots (Figure 1A), we defined two patient subgroups: one group with an exceptional good outcome (defined as ‘Good’: OS and DFS > 50 months, n = 17), and one group with an exceptional poor outcome (defined as ‘Bad’: OS < 19.5 months and DFS < 7 months, n = 47) (Figure 1B).

### Whole-genome expression analysis

Only representative snap-frozen PDAC material- defined as a minimum of 30% cancer cells on H&E staining – was used for RNA extraction. In order not to exclude tumour microenvironment for gene expression analysis, samples were used without microdissection. Total RNA was extracted using Trizol (Invitrogen, Grand Island, NY) and the RNeasy mini kit (Qiagen, Venlo, The Netherlands) according to the manufacturer’s guidelines. RNA concentration and purity were determined spectrophotometrically using the Nanodrop ND-1000 (Nanodrop Technologies, Wilmington, DE) and RNA integrity was assessed using a Bioanalyser 2100 (Agilent Technologies, Santa Clara, CA). Only samples with a RIN of at least 7.1 were used for further microarray analysis at the VIB Nucleomics Core (http://www.nucleomics.be).

Per sample, an amount of 100 ng of total RNA spiked with bacterial RNA transcript positive controls (Affymetrix) was amplified and labelled using the GeneChip 3' IVT express kit (Affymetrix). All steps were carried out according to the manufacturer’s protocol. A mixture of purified and fragmented biotinylated aRNA and hybridisation controls (Affymetrix) was hybridised on Affymetrix HG U133 Plus 2.0 arrays followed by staining and washing in a GeneChip® fluidics station 450 (Affymetrix) according to the manufacturer’s procedures. To assess the raw probe signal intensities, chips were scanned using a GeneChip® scanner 3000 (Affymetrix).

The RMA procedure was used to normalize data within arrays (background correction and log^2^-transformation) and between arrays (quintile normalization) (affy_1.22.0 package of Bioconductor) [14,15]. The MAS 5.0 algorithm (Microarray suite user guide, version 5; Affymetrix 2001) was used to assess detection above background. All probesets had a good signal and were used for further analysis. Four experimental designs were analysed: the effect of PDAC patients with a good outcome (‘Good’) *versus* surrounding pancreatic tissue (defined as ‘control’), the effect of PDAC patients with a poor outcome (‘Bad’) *versus* surrounding pancreas, the effect of ‘Bad’ *versus* ‘Good’ and the effect of all PDAC samples, irrespective of outcome, *versus* metastatic disease in the liver or peritoneum . The limma package from Bioconductor was used to assess the contrast in each experiment [16]. Statistical significance of this contrast was tested with a moderated t-test (implemented in limma). Differentially expressed genes were defined as genes with an uncorrected p-value of p < 0.001 in combination with >2 fold-change. Classical schemes to adjust for multiple testing can result in low statistical power for microarray studies . The stringent cut-off of p < 0.001 was used as an alternative, pragmatic approach to balance the number of false positives and false negatives [17].

Metastatic samples (LM and PM) were contaminated with respectively normal liver and peritoneal tissue, reflecting in upregulation of liver- and peritoneal specific genes. Therefore only genes that were not differentially expressed between LM and PM samples, considered as metastatic specific genes, were used for analysis between primary tumour and metastatic tissue.

All gene expression data will be available from the Gene Expression Omnibus (GEO, http://www.ncbi.nlm.nih.gov/projects/geo/).

Functional pathway analysis on differentially expressed probe sets was done with the Ingenuity Pathway Analysis (IPA) program (Ingenuity Systems, http://www.ingenuity.com; Redwood City, CA). For each experiment, probe sets with a corrected p-value <0.001 and a >2 fold change were used as input. If multiple probes referred to the same molecule, the average of the log-ratio values was taken for further analysis. Generated networks were ordered by a score meaning significance, estimated as the ratio of the number of input probes that map to the pathway divided by the total number of pathway probes. Significance of biological functions and canonical pathways were tested by the Fisher’s exact test p-value after application of Benjamini- Hochberg method of multiple testing correction. Significant pathways were chosen as p < 0.05, except for the significant canonical pathways in the *‘Good’ versus control* experiment where a more stringent p-value (p < 0.01) was chosen to eliminate possible false-positive results due to the large number of differentially expressed probe sets.

For each experiment, additional KEGG (Kyoto Encyclopedia of Genes and Genomes) pathway analysis was performed on up- or downregulated genes (corrected p-value <0.001 and a fold change of respectively >2 and <2) using GENECODIS, a web-based tool for enrichment analysis (http://genecodis.dacya.ucm.es ) using the NCBI Entrez Gene database [18]. Two statistical tests are implemented: the hypergeometric distribution and the χ^2^ test of independence. A stimulation-based correction approach is used to adjust for multiple testing.

## Results

### Sample selection

**Figure 2:**
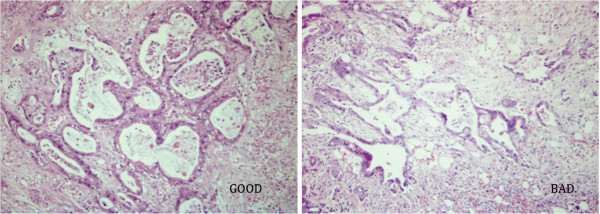
**Pathological features from ‘Good’ and ‘Bad’ patients.** Despite distinct survival data, H&E staining on formalin fixed sections from patients from the ‘Good’ outcome group (left) was similar as those from the ‘Bad’ outcome group (right). A representative sample was shown. Original magnification 100x.

Based on the definition of the 2 diverse survival outcome groups and the required RIN values above 7.1, finally 7 ‘Good’ and 10 ‘Bad’ patient samples with similar pathological characteristics remained available for gene expression analysis (Table 1, Figure 2). The median age was 61 and 67 years, respectively. All patients had negative resection margins on histopathological examination.

**Table 1:** Clinicopathological parameters of patients, with respectively good and bad outcome

**Category**	**Gender**	**Age**	**Location**	**pG**	**pT**	**pN**	**pM**	**pR**	**PNI**	**LVI**	**VI**	**Postop**	**OS**	**DFS**
GOOD	F	55	Head	2	2	0	0	0	1	0	1	0	156.4	156.4
GOOD	M	32	Head	3	3	1	0	0	1	1	0	RCT	127.9	127.9
GOOD	M	78	Head	1	3	0	0	0	0	1	0	0	71.5	71.5
GOOD	M	53	Head	3	3	1	0	0	1	0	1	RCT	67.2	67.2
GOOD	F	61	Head	3	3	0	0	0	1	0	1	0	56.4	56.4
GOOD	F	62	Head	3	3	1	0	0	0	0	1	RCT	62.7	62.7
GOOD	M	68	Tail	3	2	0	0	0	1	0	1	CT	51.5	51.5
BAD	F	75	Head	3	3	0	0	0	1	0	0	0	9.4	5.2
BAD	M	72	Head	2	3	1	0	0	1	1	1	CT	12.6	5.6
BAD	M	52	Head	3	3	0	0	0	1	0	1	0	8.4	4.1
BAD	F	78	Head	2	3	1	0	0	1	1	1	0	9.9	3.6
BAD	M	59	Head	3	3	1	0	0	1	0	0	0	6.3	2.8
BAD	F	51	Head	3	3	0	0	0	0	0	0	CT	19.4	6.5
BAD	M	74	Tail	3	1	1	0	0	1	1	1	CT	12.3	0.5
BAD	M	50	Head	2	2	1	0	0	1	1	1	CT	9.4	7.0
BAD(M)	M	67	Head				1					CT	8.3	/

*F*: female; *M*: male; *pG*: pathological tumour grade; *pT*: pathological tumour size; *pN*: pathological lymph node status; *pM*: pathological metastasis; *pR*: pathological resection margin; *PNI*: perineural invasion; *VI*: vascular invasion; *LVI*: lymphovascular invasion; *RCT*: radiochemotherapy; *CT*: chemotherapy; *OS*: overall survival; *DFS*: disease-free survival.

Additionally, 6 surrounding non-tumoural pancreatic control samples, 7 LM and 4 PM fulfilled the quality criteria and were used for microarray analysis.

### Gene expression profiling of ‘Good’ PDAC versus control

Analysis of ‘Good’ *versus* control samples revealed 3265 differentially expressed probe sets, of which 2806 could be mapped to genes in the Ingenuity Knowledge Base. IPA analysis generated networks, including ‘Cell morphology’, with *TGFβ1* (fold 2.6, p < 0.001) central to this network. ‘Cancer’, ‘Cellular growth and proliferation’, ‘DNA repair’, and ‘Cellular movement’ were differentially expressed functions. Differentially expressed canonical pathways (p < 0.01) are shown in Table 2. The Integrin pathway (including Integrin β4 (*ITGB4*): fold 5.5, Integrin β5 (*ITGB5*): fold 5.9, and Integrin α6 (*ITGA6*): fold 4.6; all p < 0.001) was most significant, followed by the Ephrin pathway (including Ephrin receptor A2 (*EPHA2*): fold 5.9, Ephrin receptor B2 (*EPHB2*): fold 3.3, Ephrin A1 (*EFNA1*): fold 3.4, Ephrin A4 (*EFNA4*): fold 2.0 and Ephrin B2 (*EFNB2*): fold 3.4; all p < 0.001). KEGG pathway analysis of genes overexpressed in ‘Good’ samples showed upregulation of elements of the p53 signalling, Wnt/β-catenin signalling, Notch, MAPK, and Hedgehog signalling pathways (Table 2).

**Table 2:** Differentially expressed canonical pathways (IPA) and upregulated KEGG pathways (GENECODIS) in ‘Good’ and ‘Bad’ PDAC

	**Good*versus*control**		**Bad*versus*control**	
**Canonical pathways**^**a**^	P-value	Upregulated genes^c^	P-value	Upregulated genes^c^
Integrin signalling	5.62^E^-7	RAC1, RAC2, ITGB4, ITGB5, ITGA6, ACTN1, MAP2K2, GSK3B, PPP1R12A, ARF1, ACTG2	4.79^E^-6	RAC1, ITGA2, ITGA3, ITGA6, ITGB1, ITGB4, ITGB5, ITGB6, ACTN1, ARF1
Ephrin receptor signalling	0.00002	RAC1, RAC2, EPHA2, EPHB2, EFNA4, EFNB2, MAP4K4, MAP2K2, STAT3, RHOA, ADAM10, VEGFA	0.00001	RAC1, EFNA5, EFNB2, EPHA2, EPHB4, STAT3, ADAM10, FGF1, VEGFA, PDGFC
Molecular mechanism of cancer	0.00063	RAC1, RAC2, CCND1, MAP2K2, TGFβ1, GSK3B, BRCA1, CDH1, BMP2, SMAD6, BAX, CTNNB1		
P53 signalling	0.00089	TP53, PIK3C2A, RAC1, BAX, BIRC5, SERPINB5, GSK3B, BRCA1	0.02757	PRKDC, RAC1, BAX, CCND1, BIRC5, SERPINB5, CTNNB1, CDK2
Wnt/β-catenin	0.00550	RAC2, CSNK1A1, CSNK1E, SOX9, TGFβ1, SOX4, LRP5, CTNNB1, WNT10A	0.00323	CSNK1A1, TGFβ1, DKK1, DKK3, WNT5A, WNT10A, SOX4, SOX11, TCF7L2, TCF3
Pancreatic adenocarcinoma			0.00776	JAK1, RAC1, STAT3, CCND1, BIRC5, VEGF, TGFβ1, ERBB2, CDK2
PI3K/AKT Signaling	0.00933	RAC1, RAC2, JAK1, MAP2K2, PPP2R5		
**KEGG pathways**^**b**^				
P53 Signaling	2.20^E^-12	TP53, CDKN6, CCND1, CDK1, CDK2, SFN	3,03^E^-8	CDK1, CDK2, BAX, SERPINB5, CCND1, SFN
Wnt signalling	2,67^E^-07	WNT10A, CTNNB1, CTBP1, LRP5, TCF7L2, FZD8, GSK3B, PPP3R1, RAC1	0.00011	WNT5A, WNT10A, DKK1, DVL1, CTNNB1, CSNK1A1, CSNK1E, LRP5, RAC1, TCF7L2
Pancreatic cancer	3.00^E^-6	TGFβ1, RAC1, JAK1, VEGFA, ERBB2, STAT3,TP53, RAC2	0.00001	RAC1, TGFβ1, TGFα, VEGFA, ERBB2, STAT3, RAD51
NOTCH signalling	2.40^E^-6	JAG1, HES1, CTBP1, CTBP2, ADAM10	0.00012	DVL1, HES1, CTBP1, ADAM10
MAPK signalling	0.00015	FGFR2, TGFβ1, MAP2K5, MAP2K2, MAP2K3, MAP2K7, RAC1, DUSP10, DUSP3		
Hedgehog signalling	0.00836	CSNK1E, BMP2, GSK3B, CSNK1A1		

^a^IPA was performed on respectively 2.806 (good) and 1.692 (bad) differentially expressed probe sets (with entry in the Ingenuity Knowledge Base; http://www.ingenuity.com). The most significant networks, functions and canonical pathways are listed.

^b^ KEGG analysis was performed on respectively 2.033 and 1.285 probesets upregulated in the good and bad PDAC samples using GENECODIS.

^c^ A selection of upregulated genes contributing to the pathways, is given.

### Gene expression profiling of ‘Bad’ PDAC versus control

Microarray analysis comparing ‘Bad’ *versus* control samples defined 1905 differentially expressed genes. IPA analysis on 1692 mapped genes generated networks, such as the network related to ‘Drug metabolism’, including *TGFβ1* (fold 2.4) and *LOXL2* (fold 3.9), (p < 0.001). Similar to the *‘Good’ versus control* comparison, the functions ‘Cancer’, ‘Cellular growth and proliferation’ and ‘Cellular movement’ were differentially expressed, but with even higher fold changes. Analysis of canonical pathways also revealed the Integrin pathway as most significant (including *ITGA2*: fold 5.0, *ITGA3*: fold 3.1, *ITGA6*: fold 5.3, *ITGB1*: fold 2.0, *ITGB4*: fold 5.8, *ITGB5*: fold 5.0 and *ITGB6*: fold 5.4; all p < 0.001), on top of the Ephrin receptor signalling (including *EPHA2*: fold 7.3, *xEPHB4*: fold 2.0, *EFNA5*: fold 3.9 and *EFNB2*: fold 3.0; all p < 0.001), the Wnt/β-catenin pathway and pancreatic adenocarcinoma signalling (Table 2). Genes involved in the p53 signalling pathway, the Wnt/β-catenin and the Notch signalling were highly upregulated (Table 2) in ‘Bad’ PDAC samples (KEGG analysis, GENECODIS).

### Molecular characteristics of ‘Bad’ versus ‘Good’ PDAC

**Figure 3:**
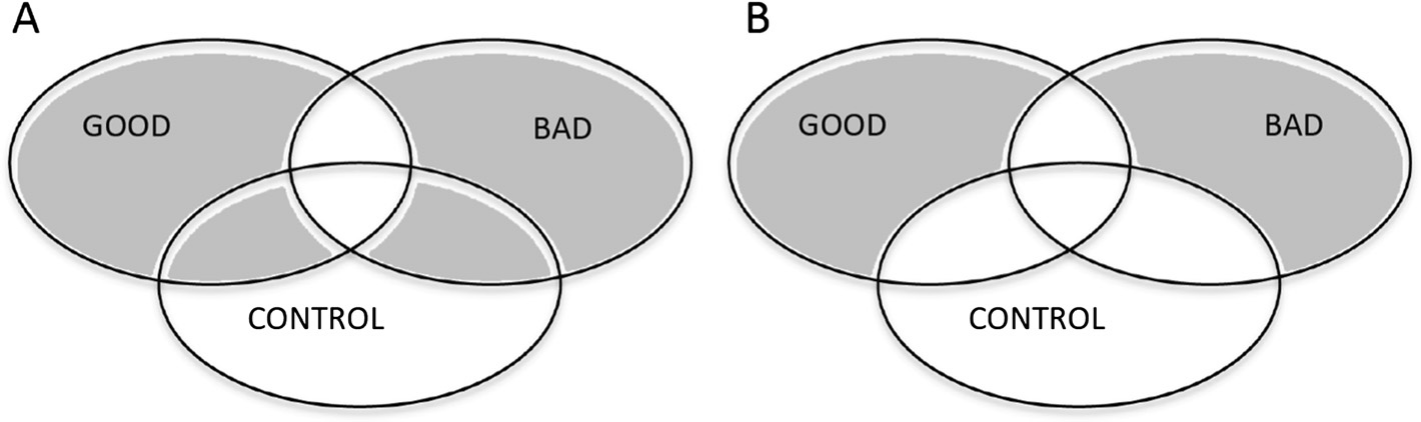
**Molecular characteristics of ‘Bad’ vs. ‘Good’ PDAC.** (**A**) First, genes differentially expressed between the ‘Good’ and the ‘Bad’ PDAC samples were used for IPA analysis. (**B**) Secondly, we compared genes differentially expressed between the *‘Good’ versus control* and the *‘Bad’ versus control* analysis to exclude pancreas-related genes. The control samples in both experiments were the same.

To study gene expression profiling related to poor outcome, we first studied differentially expressed genes between ‘Bad’ and ‘Good’ PDAC samples (Figure 3A). A total of 131 genes were differentially expressed, i.e. 69 upregulated and 62 downregulated genes in ‘Bad’ PDAC (Table 3). The networks ‘Cell morphology’ (including *SNAI2* (fold 2.9) and *TGFβR1* (fold 3.3); p < 0.001), ‘Cell signalling’ and ‘Cellular movement’ were generated from differentially expressed genes (IPA). No cancer-related canonical pathways or KEGG pathways were differentially expressed between both PDAC groups.

**Table 3:** **Top 15 of differentially expressed genes, between bad*versus*good outcome PDAC samples**

**Gene Symbol**	**Gene name**	**Fold bad/good**	**P-value**
CPB1	Carboxypeptidase B1	31.03	3.16^E^-05
CTRB2	Chymotrypsinogen B2	24.38	2.78^E^-05
PLA2G1B	Phospholipase A2, group IB, pancreas	20.35	0.00022
PNLIPRP2	Pancreatic lipase-related protein 2	19.48	0.00019
PNLIP	Pancreatic lipase	19.06	0.00048
CEL	Carboxyl ester lipase (bile salt-stimulated lipase)	18.89	0.00011
CPA1	Carboxypeptidase A1, pancreatic	18.57	6.68^E^-05
CELA3A	Chymotrypsin-like elastase family, member 3A	17.10	2.47^E^-05
CELA3B	Chymotrypsin-like elastase family, member 3B	16.56	2.01^E^-05
CPA2	Carboxypeptidase A2 (pancreatic)	14.43	0.00016
CLPS	Colipase, pancreatic	11.55	0.00035
CTRC	Chymotrypsin C (caldecrin)	11.17	0.00023
KRT6A	Keratin 6A	10.23	0.00090
PRSS2	Protease, serine, 2 (trypsin 2)	8.87	0.00092
DEFA5	Defensin, alpha 5, Paneth cell-specific	−13.95	9.04^E^-08
SLC26A3	Solute carrier family 26, member 3	−13.76	4.08^E^-08
SI	Sucrase-isomaltase (alpha-glucosidase)	−8.95	2.29^E^-07
TAC3	Tachykinin 3	−8.06	0.00029
PRSS7	Protease, serine, 7 (enterokinase)	−6.93	1.99^E^-08
DEFA6	Defensin, alpha 6, Paneth cell-specific	−6.50	1.50^E^-06
VIP	Vasoactive intestinal polypeptide	−6.12	1.82^E^-05
RBP2	Retinol binding protein 2, cellula	−5.68	1.72^E^-07
UGT2B17	UDP glucuronosyltransferase 2 family, polypeptide B17	−5.33	0.00090
CDH19	Cadherin 19, type 2	−4.90	0.00089
SYNM	Synemin, intermediate filament protein	−4.86	1.53^E^-05
FOXA1	Forkhead box A1	−4.30	6.00^E^-07
CLCA1	Chloride channel accessory 1	−3.90	2.05^E^-05
ELF5	E74-like factor 5	−3.74	1.50^E^-06
AKR1C1	Aldo-keto reductase family 1, member C1	−3.63	0.00043

Next, we analysed differentially expressed genes between the *‘Good’ versus control* and the *‘Bad’ versus control* experimental designs to exclude pancreas-related genes (Figure 3B). Only genes from the MAPK and Hedgehog signalling pathways were strongly expressed in the ‘Good’ samples (GENECODIS). Genes involved in Pancreatic cancer signalling pathway, p53 signalling, Wnt/β-catenin and Notch signalling were expressed in all PDAC samples, but the constitutive genes varied. ‘Bad’ samples overexpressed the Wnt signalling molecules *DKK1* (fold 7.9), *Wnt5a* (fold 3.6) and *DVL1* (fold 2.8)(p < 0.001), whereas *FZD8* (fold 2.7, p < 0.001) and *GSK3B* (fold 2.0, p < 0.001) were only upregulated in ‘Good’ samples. *TP53* was only overexpressed in the ‘Good’ group (fold 2.7, p < 0.001).

### Identification of metastasis-associated genes

After excluding liver- and peritoneum specific genes, 358 genes were differentially expressed between the primary tumour and the metastatic samples. Of these genes, 278 were upregulated in primary PDAC and 80 were upregulated in metastatic tissue. Multiple networks and functions were generated from differentially expressed genes (IPA), including ‘Cancer’, ‘Cell signalling’, and ‘Cell cycle’. The ‘Human embryonic stem cell pluripotency’ and Wnt/β-catenin canonical pathways were significant. KEGG pathway analysis (GENECODIS) revealed expression of genes from the TGFβ and Wnt/β-catenin pathways in primary PDAC and expression of the TGFβ pathway-related genes in metastatic tissue (Table 4). To discover pathways potentially contributing to the metastatic process, we looked for genes upregulated in the *PDAC versus control* experiments (‘Good’ *versus* control and ‘Bad’ *versus* control) and in the *Metastases versus PDAC* comparison. In total 29 genes met these criteria, including *β-catenin, ANP32A, HPGD, SET* and *SP1* (fold change between metastases *versus* PDAC respectively 3.0, 3.4, 2.5, 3.6 and 2.0; all p < 0.001) ( Additional file 1: Table S1).

**Table 4:** Upregulated KEGG pathways (GENECODIS) in primary PDAC and metastatic PDAC samples

	**PDAC*versus*Metastases**		**Metastases*versus*PDAC**	
KEGG Pathway^a^	P-value	Upregulated genes^b^	P-value	Upregulated genes^b^
Wnt signalling	0.00969	FZD1, FZD10, WNT5A, CCND2		
TGFβ pathway	0.00574	LTBP1, THBS4, MBPR1B	0.00100	SP1, PPP2R1B, ACVR1C

^a^ KEGG analysis was performed on respectively 278 and 80 genes upregulated in the PDAC and metastases samples using GENECODIS.

^b^ A selection of upregulated genes contributing to the pathways, is given.

## Discussion

Unravelling the molecular characteristics of pancreatic cancer is crucial for a better understanding of the tumour biology in order to develop novel therapeutic strategies. Correlation of gene expression profiles with patient survival might detect genes and pathways that drive PDAC invasiveness as clinicopathological parameters alone seem not sufficient to explain the variability in survival after curative resection. Therefore, in the present study, we performed whole genome expression analysis of 2 subgroups of patients with extremely diverging overall and disease-free survival rates, despite having similar clinicopathological features.

In contrast to previous studies that used microdissection or fine needle aspiration techniques to enrich the samples for neoplastic cells [11,19,20], we used whole-tumour samples with the aim not to exclude the tumour micro-environment even though discrimination between tumoural and environmental RNA is technically impossible in whole-tumour samples. On the other hand, PDAC is characterized by an abundant desmoplastic stromal reaction, which plays an important role in tumorigenesis, tumour progression, and therapy resistance [12,13]. Indeed, increasingly new therapeutic regimens are studying agents that aim to target the desmoplastic stromal reaction [21-23]. Therefore, in order to keep the molecular information of the microenvironment but to reduce background RNA contamination, we used high-quality snap-frozen samples with a pathologically proven minimum of 30% cancer cells. This approach led to a small but still representative sample size for microarray analysis.

In our study, the Integrin and Ephrin pathways were upregulated in all PDAC samples, irrespective of outcome. These pathways were not highlighted in studies on microdissected PDAC [11]. Both pathways appear to play an important role in the interaction between cancer cells and the surrounding stroma. The Integrin family of cell adhesion receptors has been implicated in tumour progression as they contribute to the interplay between tumour and micro-environment by binding directly to components of the extracellular matrix (ECM) [24]. Due to the abundance of ECM, the integrin-mediated cell adhesion signalling may play an important role in PDAC tumour growth, migration and even in therapy resistance [25,26]. Various integrins, such as *ITGA6, ITGB4* and *ITGB5*, are upregulated in ‘Good’ and/or ‘Bad’ PDAC samples*.* In cell culture studies, *ITGB1* has been shown to play a critical role in pancreatic cancer progression and in metastasis in particular [27,28]. Upregulation of *ITGB1* in ‘Bad’ PDAC, might highlight its potential therapeutical impact.

Ephrin receptors are similarly promising therapeutical targets as they mediate cell-cell interactions both in tumour cells and in the tumour micro-environment, and thereby may affect tumour growth, invasiveness, angiogenesis, and metastasis [29]. *EPHA2*, related to poor clinical outcome in PDAC, has already been successfully investigated as target in PDAC cell lines [30,31]. Indeed, in our study, *EPHA2* was highly upregulated as PDAC with poor outcome, supporting its potential clinical relevance.

Embryonic signalling pathways are known to play a role in both the tumoural and the stromal compartment and in different stages of PDAC [32]. Hedgehog signalling (Shh) e.g. has been implicated in the initiation of PDAC, and was overexpressed in PDAC samples with good overall survival in our series [33,34]. The Wnt/β-catenin pathway seems to be involved in a later stage of PDAC tumorigenesis [9,34,35]. In our study, elements from the canonical Wnt/β-catenin pathway were upregulated in all PDAC samples. However, in patients with poor survival, genes from both the canonical and non-canonical pathway, including *Wnt5A* and *DVL1*, were upregulated [35,36]. The expression of *Wnt5A* has already been shown to be induced in PSC [35]. Upregulation of *DKK1*, a Wnt/β-catenin pathway antagonist, may promote tumour invasiveness though the exact mechanism is yet unknown [37].

Overexpression of Notch signalling in PDAC correlates with tumour proliferation and migration [38]. Notch has been shown to regulate pancreatic cancer stem cells and would have a role in the acquisition of epithelial-mesenchymal transition (EMT) by inducing *SNAI2* expression due to *JAG1* overexpression [39,40]. Although *JAG1* was upregulated in all our PDAC samples irrespective of survival, *SNAI2* was upregulated in the *‘Bad’ versus ‘Good’* PDAC samples. The upregulation of many EMT-related genes, such as *TGFβR1, FGFBP1TGFβ1LOXL2, TWIST1* and *Wnt5A*, and the downregulation of *FOXA1* in the *‘Bad’* PDAC samples might support the role of EMT in the aggressiveness of PDAC [41]. Additionally, upregulation of *MALAT1* in the ‘*Bad’* samples may suggest this gene to be further explored as it is upregulated in many other tumours too and associated with cancer metastasis and recurrence [42,43].

Finally we identified a PDAC metastasis-related genetic profile containing 358 differentially expressed genes between the primary tumour and metastatic tissue. Molecular knowledge on the metastatic process in PDAC is currently lacking and the published data are inconsistent [9,44-46]. Moreover, the majority of studies are based on cell lines, xenograft models and rapid autopsy material. In the current study, we used fresh human samples of both liver and peritoneal metastases. In order to focus on metastasis-specific genes, we excluded tissue-associated genes, i.e. genes that were differentially expressed between liver and peritoneal tissue samples. However, in this way, we might also have excluded metastasis-specific genes. In our study, 358 genes were differentially expressed, including genes related to the Wnt/β-catenin pathway and the TGFβ pathway. Comparing our differentially expressed genes with metastatic genes described in other studies, only 7 genes overlapped (*COMP, PCDH7, PTP4A1, CXCR4, NR4A3, ANGPT1 and TIMP3*) [9,44-47]. A total of 29 genes were upregulated in metastases as compared to primary PDAC and control samples. One of these genes, *β-catenin*, may deserve further study because of several reasons. *β-catenin* has a role in tumorigenesis as an essential transcriptional co-activator in the canonical Wnt pathway, but it also plays a critical role in cadherin-based cell-cell adhesion [48]. *β-catenin* seems also to be a major determinant in EMT and in the reverse mesenchymal to epithelial transition (MET), necessary for cells to home in distant organs. Furthermore, *β-catenin* mediates transcription of MMP that degrade the ECM [49]. Our results support further investigation of its role in PDAC progression. Another gene, *SP1* is linked with *STAT3* and hence would regulate metastasis [50].

Limitations of the current study are the rather small sample size and the lack of clinical validation of our findings. These 2 concerns however, seem hard to overcome since PDAC is a rare disease of which good quality tissue is difficult to obtain. Additionally, PDAC has an abundant desmoplastic reaction that is overwhelmingly represented as compared to cancer cells, making many human tissue samples not representative. Microdissection of cancer cells might be an alternative to study PDAC, although this technique has its own inherent limitations, such as its technical difficulty and consequently its time-consuming activity, and the problem of RNA degradation [51]. Moreover, we believe that the only way to study human PDAC as a whole entity is to include its microenvironment in the analyses, especially since the latter has been shown to play a crucial role in tumour invasiveness and progression. The data from our current study might therefore provide valuable results with respect to gene expression and pathways involved in PDAC. Nonetheless, before these genes or pathways might be used as potential therapeutic targets in clinical setting, they need to be validated first either in a large number of human PDAC samples or in preclinical animal experiments.

## Conclusion

The Integrin and Ephrin pathways seem to play an important role in pancreatic carcinogenesis and progression, including *ITGB1* and *EPHA2* as most important players. The Wnt/β-catenin pathway and EMT might additionally contribute to PDAC progression and metastasis, with *β-catenin* as a central mediator. Further validation of the role of these genes and pathways is needed.

## Abbreviations

PDAC: Pancreatic ductal adenocarcinoma; OS: Overall survival; DFS: Disease-free survival; EMT: Epithelial-mesenchymal transition; LM: Liver metastasis; PM: Peritoneal metastasis; H&E: Haematoxylin-Eosin; CI: Confidence interval; RIN:RNA: Integrity number; IPA: Ingenuity pathway analysis; KEGG: Kyoto encyclopedia of genes and genomes; ECM: Extracellular matrix.

## Competing interests

The authors declare that they have no competing interests.

## Authors’ contributions

AVDB designed and performed the study, analysed the data and wrote the manuscript. HV participated in drafting the manuscript. RVE has been involved in analysing the data. OG contributed to data collection and data analysis and revised the manuscript. BT conceived and designed the study, interpreted the data and wrote the manuscript. All authors read and approved the final manuscript.

## Supplementary Material

Additional file 1 **Table S1.** Selection of 29 genes, upregulated in *‘Good versus control’***,***‘Bad versus control’* and *‘Metastases versus Pancreatic cancer (PDAC)’.* Click here for file
