# Supplementary material for: Molecular markers associated with outcome and metastasis in human pancreatic cancer
Source: J Exp Clin Cancer Res. 2012 Aug 27;31(1):68. doi: 10.1186/1756-9966-31-68 (PMC3511800; doi:10.1186/1756-9966-31-68)
Supplement: Additional file 1 — Table S1. Selection of 29 genes, upregulated in ‘Good versus control’,‘Bad versus control’ and ‘Metastases versus Pancreatic cancer (PDAC)’. [file 1756-9966-31-68-S1.docx]

Supplementary Table 1. Selection of 29 genes, upregulated in *‘Good versus control’*, *‘Bad versus control’* and *‘Metastases versus Pancreatic cancer (PDAC)’*

| **Gene Symbol** | **Gene name** | **Fold M vs. PDAC** | **P-value** |
| --- | --- | --- | --- |
| SET | SET nuclear oncogene | 3.64 | 3.43^E^-11 |
| ANP32A | Acidic (leucine-rich) nuclear phosphoprotein 32 family, member A | 3.38 | 9.45^E^-14 |
| TMED2 | Transmembrane emp24 domain trafficking protein 2 | 3.03 | 6.49^E^-11 |
| CTNNB1 | β-catenin | 2.98 | 2.57^E^-11 |
| RPL37A | Ribosomal protein L37A | 2.96 | 6.36^E^-12 |
| SLC16A1 | Solute carrier family 16, member 1 | 2.93 | 1.46^E^-07 |
| ATP6VOE1 | ATPase, H+ transporting, lysosomal 9kDa, V0 subunit e1 | 2.60 | 2.18^E^-12 |
| ENO1 | Enolase 1 | 2.57 | 3.35^E^-06 |
| THOC4 | THO complex subunit 4 | 2.57 | 5.21^E^-08 |
| MSH6 | MutS homolog 6 | 2.52 | 3.23^E^-10 |
| MEX3D | Mex-3 homolog D | 2.51 | 7.42^E^-06 |
| SPTBN1 | Spectrin, beta, non-erythrocytic 1 | 2.48 | 1.67^E^-11 |
| HPGD | Hydroxyprostaglandin dehydrogenase 15-(NAD) | 2.45 | 0.00014 |
| EIFA5 | Eukaryotic translation initiation factor 5A | 2.42 | 2.38^E^-08 |
| AK3L1 | Adenylate kinase 3 | 2.41 | 9.27^E^-06 |
| AP1S1 | Adaptor-related protein complex 1, sigma 1 subunit | 2.31 | 3.69^E^-07 |
| VDAC1 | Voltage-dependent anion channel 1 | 2.23 | 1.56^E^-08 |
| PRKDC | Protein kinase, DNA activated, catalytic polypeptide | 2.20 | 2.01^E^-06 |
| ARPC4 | Actin related protein 2/3 complex, subunit 4, 20kDa | 2.15 | 3.52^E^-08 |
| AMFR | Autocrine motility factor receptor, E3 ubiquitin protein ligase | 2.15 | 7.04^E^-07 |
| YWHAZ | Tyrosine 3-monooxygenase/tryptophan 5-monooxygenase activation protein | 2.12 | 3.09^E^-06 |
| SDHC | Succinate dehydrogenase complex, subunit C, integral membrane protein, 15kDa | 2.09 | 8.05^E^-08 |
| APLP2 | Amyloid beta (A4) precursor-like protein 2 | 2.05 | 1.46^E^-07 |
| SP1 | Sp1 transcription factor | 2.04 | 7.47^E^-07 |
| CAMK2N1 | Calcium/calmodulin-dependent protein kinase II inhibitor1 | 2.03 | 0.00035 |
| STT3A | STT3, subunit of the oligosaccharyl- transferase complex, homolog A | 2.03 | 7.71^E^-08 |
| HSPD1 | Heat shock 60kDa protein 1 | 2.01 | 2.18^E^-06 |
| ENSA | Endosulfine alpha | 2.01 | 1.26^E^-08 |
| NUTF2 | Nuclear transport factor 2 | 2.00 | 9.26^E^-08 |
